# Atlanta Society of Medicine

**Published:** 1890-07

**Authors:** 


### Society Notes.

### ATLANTA SOCIETY OF MEDICINE.

*Stated Meeting, June 3d, 1890.*

F. W. McRAE, M. D., Vice-President, in the Chair.

### DIVISION OF STRICTURE OF THE URETHRA WITHOUT SUBSEQUENT SOUNDING OR DILATATION.

On the call for reports of cases, the following remarks were made by Dr. J. McF. Gaston:

Having on a previous occasion reported a case of aspiration of the bladder and subsequent external urethrotomy from complete occlusion of the urethra, a further history of the case will be likely to interest the Society.

Being called to the same patient in April, he was found suffering from urinary infiltration into the scrotum and adjacent tissues, with quite constitutional disturbances. Notwithstanding the continuation of free flow of urine through the perineal opening, the urine had been forced into this abscess cavity in advance of it, and not having an outlet anteriorly through the penis, it percolated into the cellular structure by infiltration, producing its usual results. He was ordered internally the following: ℞ Nitrate of potash, ʒi; calomel, grs. vi; tartar emetic, gr. i., divided in six powders—one to be taken every two hours. An external application was made as follows: ℞ Mercurial ointment, belladonna ointment, aa ℥ss; pulv. camphor, ʒi. Mix and smear over the scrotum and neighboring parts with cotton and a supporting bandage.

Some relief being afforded, the patient returned shortly afterwards to the Providence Infirmary, when a free outlet through the fistulous opening was secured by an incision, and ultimately it was found that a small amount of urine could be forced through the penile urethra. At this stage Dr. Elkin succeeded in passing a filiform whalebone bougie through the stricture into the bladder.

By careful manipulation with the staff-loop of Maisoneuve's urethretome, it was guided into the bladder, and the blade was used to divide the stricture up to the point of admitting a No. 32 sound with facility.

It was thought best, in view of all the past history of this case, not to follow up with the use of the sound, and this operation being done on May 1st, there was no repetition of the sounding, or other measure for dilatation, until the 25th of the month. The urine had been passing freely during this period by the perineal fistulas by the urethra, but it was deemed expedient to verify the caliber of the urethra preparatory to discharging the patient from the infirmary.

A 4 per cent. sol. of cocaine was injected into the canal, and upon introducing a No. 12 English catheter, it was arrested a little in advance of the perineal fistula. It occurred to me that it might have lodged in a pocket resulting from the former abscess of this part, and it was suggested to Dr. Elkin to pass a large female catheter through the fistulous opening forward, which was passed readily until it came in contact with the catheter in the penile urethra. By withdrawing the male catheter until the end of it rested upon the end of the female catheter, and thus urging the former along the line taken by it, there was no further difficulty in reaching the bladder. A No. 26 sound was then introduced, and subsequently a No. 30 passed without difficulty, indicating a caliber entirely satisfactory, so that the patient may now be discharged.

The practical question as to the utility of passing the sound for the purpose of preventing the union of the margins of a divided stricture, is well presented in this case. It affords conditions likely to satisfy impartial observers that when a constricting elastic band, forming a stricture of the urethra, is completely divided at one point, there is no necessity for dilatation by bougies or sounds. If the incision passes entirely through all the fibres of this ring, it is evident that the elasticity of the tissue must draw the edges apart, and any means of dilatation which may be subsequently employed acts upon the intervening cellular tissue without distending the proper components of the gaping indurated stricture. We should naturally expect the gradual absorption of the components of the divided band if left to rest, whereas, by the initiation of bougies or sounds this process would be materially retarded, if not prevented.

Under the very unfavorable conditions of this case, it is found that after the lapse of twenty-five days, without the introduction of a sound or bougie, the caliber of the urethra is preserved so as to afford a full flow of urine, and the result warrants the repetition of this procedure by those who may have to operate on close strictures of the urethra.

CONSTIPATION.

Dr. Baird reported the case of a young lady, recently married, which had given him considerable anxiety for several days, notwithstanding the absence of all local and general symptoms of a grave character.

The facts were about these:

After several days of rather unnatural freedom of the bowels—the stools showing incomplete digestion—two days elapsed without any action. On Tuesday night she took, on her own responsibility, five grains of calomel. Wednesday night, as the bowels had not acted, she took two grains more without effect. Thursday, she took a seidlitz powder and Thursday night a large dose of castor oil, with negative results. Friday, becoming alarmed, she consulted him. Found no fever, no abdominal pain or tenderness, and no tympaniti—no nausea or vomiting. Tongue moist and clean—no hiccough. Ordered restricted and liquid diet, a seidlitz powder and a bottle of solution of citrate of magnesia during the afternoon, to be repeated, if necessary, next morning, and to be followed in the absence of copious evacuations, by a simple enema. To his surprise he found on Saturday, notwitstanding, the faithful execution of his instructions, and the voluntary addition, on the part of the patient, of several cathartic pills, no movement had been secured. He then directed that three ounces of castor oil be thrown into the rectum, and repeated in four hours, to be followed next morning by a copious warm water enema. Sunday—general condition unchanged—no fecal evacuation. Enema returned, showing the presence of some oil, but no fecal matter. Examination by the vagina and by the rectum showed no fecal impaction. He then proceeded, after consultation with Dr. Anthony, to introduce a long tube into the bowel, as high as the transverse colon, and, by means of a Davidson's syringe, attached, injected a large quantity of warm water, in which some turpentine soap had been dissolved. The injection was continued until the colon was distended and a feeling of tightness and some pain was experienced. The water was immediately expelled, deeply stained, and having a decided fecal odor and bringing away two small fecal lumps. The process was at once repeated with similar results, but without the scybalous masses. Monday—patient comfortable, but a little nervous and depressed in spirits, as no movement had occurred. Reintroduced the long tube and injected one quart of olive oil. Directed that it be retained until next morning, when a rectal injection of warm water was to be used. Tuesday—no fecal movement. General and local condition about the same. Introduced the long tube as before and threw into the transverse colon eight ounces of castor oil and two drachms of oil of turpentine. Ordered a tablespoonful of castor oil, by the mouth, every two hours until four doses were taken. After about sixteen hours, there occurred a fairly free evacuation of fecal matter of soup-like and soft, pasty consistency.

Since then the patient has done well, and has had daily stools under the influence of mild laxatives.

She was habitually constipated. The rectum was dilated and pouch-like. The injections, without the long tube, did not, apparently, go beyond the sigmoid flexure.

IMPETIGO CONTAGIOSA.

Dr. Hutchins reported a case of impetigo, or impetigo contagiosa, the latter, according to Crocker, comprising both varieties, and due to presence of "pus cocci." In bearded part of face a number of yellowish crusted lesions or "sores." When first examined, under a poor light, there was a suspicion of ringworm of the beard, and some extracted hairs were examined, but no fungus found. The next day the disease was diagnosed correctly, and antiseptic treatment continued with following ointment: ℞ Hg. bichlor., gr. v.; spt. vin. rect., ʒii.; ungt. petrolati, ℥i. M., and add to basin of water when washing the face ʒii. of ℞ Hg. bichlor., ʒiss.; spt. v. rect. et aquæ aa ℥i. M. Discharged in less than two weeks; pustules about well. There was slight vesiculation from applications. Dr. McRae had kindly referred this case to Dr. Hutchins with a history of ordinary ringworm of the neck. The treatment would have included epilation if the case had been parasitic sycosis, and the duration probably three months. The curious fact was mentioned that an ordinary ringworm would sometimes occur on the bearded face without involving the hairs. This patient had a patch suggesting such an extension. A large boil on the back of the neck might have been the source of infection for the pustules.

### BATTEY'S OPERATION.

DR. VIRGIL O. HARDON reported a case of Battey's operation, and exhibited the specimens. The patient, an unmarried woman about 20 years of age, had suffered for three years with severe and persistent pains in the pelvis, which had undermined her health to such an extent that of late she had become bedridden. All other means of cure having been exhausted without effect, Battey's operation was undertaken as a last resort. The left ovary was found to contain a small cyst, while the right was small and cirrhotic. The patient made a good recovery, her temperature at no time exceeding 101. It is yet too early to report the ultimate effects of the operation.

---

*Stated Meeting, June 17th, 1890.*

WILLIAM PERRIN NICOLSON, M. D., President, in the Chair.

### CHOLECYSTOTOMY.

Dr. Virgil O. Hardon reported a case of obstruction of the cystic duct successully treated by cholecystotomy. The patient, a woman 28 years of age, the mother of five children, had always been well until about seven years ago, when she began to suffer with attacks of "bilious colic." In the intervals between the attacks her health was good. About five years ago she first noticed a lump in the right side below the ribs, which gradually increased in size up to the present time. Her general health began to fail four years ago, and weakness and emaciation increased until she was confined to the bed nearly all the time. The appetite remained fair, the bowels were constipated. There was no jaundice at any time. She had no pain, except during the paroxysms of colic.

On examination an elastic fluctuating tumor could be felt on the right side, commencing at the lower border of the liver, and extending obliquely downward and to the left as far as the umbilicus. It was apparently about five inches long, and about two inches broad. The lower extremity could be pushed upward and to the left in a circle, of which the upper extremity of the tumor was the axis. It was tender to the touch. It was apparently attached to the liver by its upper extremity.

A diagnosis was made of distension of the gall bladder from obstruction, and it was decided to perform cholecystotomy with a view to its relief. An incision was made in the median line extending two inches above the navel and one inch below it. The distended gall bladder at once came into view. It was punctured with a trocar and half a pint of clear serum evacuated. The opening was then enlarged with scissors and a finger introduced, which came into contact with a mass of gall stones, which were removed to the number of thirty-eight. The finger was then passed down to the orifice of the cystic duct, and a stone found lying impacted in the duct with a firm constriction between it and the gall bladder. This stone could not be removed by manipulation, and therefore a bistoury was passed down to the constriction and its edges slightly nicked at various points. By further effort the stone was then dislodged and removed through the gall bladder. Examination of the cystic and common ducts showed that no other obstruction was present. The incision in the gall bladder was closed by a continued Lumbert suture of catgut, and dropped back into the abdomen. The incision into the abdominal wall was closed and dressed in the usual manner.

The patient made a good recovery without a single unfavorable symptom. Her highest temperature was 100.5, on the evening of the second day, and fell to normal on the fourth day. It is now the eleventh day, and she is convalescent.

In this operation valuable assistance was rendered by Drs. Rosser and Gwinn, of Conyers, and Dr. Gibson, of Newton county, and the rapid recovery of the patient was largely due to the faithful manner in which the after treatment was carried out by the latter gentleman.

A CASE OF LAPAROTOMY.

Dr. J. G. Earnest reported a laparotomy on a woman aged 35, with history of excessive menstruation and repeated attacks of dysentery, associated with peritonitis. The operation was urged by the patient on account of "sinking spells" that seemed to threaten life, and the failure of a number of reputable physicians to better her condition. Diagnosis was observed by presence of fluid in abdomen, perfectly fixed uterus, immobility and hardness of the vaginal vault, etc., but was believed to be a case of fibro-cystic tumor of uterus. Upon opening the abdomen there was found a tumor, apparently fibrous, springing from a pedicle on the under side of the umbilicus, and extending five or 6 inches in the direction of the hypochondrium, ending in a sharp conical point. It was not a displaced kidney, as at first thought, but apparently a fibrous mass springing from a dense pedicle about three-fourths of an inch in diameter. The abdomen was lined with a parchment-like material that did not bleed when cut. This substance was so thick as to fill up the right iliac region, concealing entirely the right ovary and parts in that neighborhood, spreading out over the uterus and left ovarian region, where it seemed to be half an inch or more thick, and completely lining the abdominal cavity up to a point above the umbilicus. At the upper portion it was drawn in and detached from the abdominal wall for an inch or more, so that the upper opening was about four inches in diameter. This free margin was about a quarter of an inch thick. That thickness was pretty uniformly maintained along the abdominal opening. Through this opening, at the top, the small intestine entered and was adherent to its walls on all sides. The intestine was very much matted together by adhesions, and very rough and dark. There were several spots visible that had a gangrenous appearance, and one that was raised, white, and seemed to be filled with smaller cells, distended with gas. The abdomen was full of clear fluid. After carefully examining the situation, it was thought best to close the wound without removing the growth or tumor, or disturbing the intestinal adhesions. On the third day a diarrhœa set in, and she continued to pass frequent and large stools until the ninth day, when the diarrhœa suddenly stopped and the abdomen began to swell. On the tenth day stitches were removed, and the usual dressing with adhesive strips applied. That night the lower angle of the wound was forced open and began discharging fecal matter. The next day she was placed on the table, etherized, and the abdomen reopened. After clearing out fecal matter two openings were found in the intestine, one about an inch and a-half long and an inch and a quarter in its transverse diameter at the widest point. The opening was very irregular in shape. Below it some inches, was a smooth circular opening about three-eighths of an inch in diameter. These were carefully closed with silk, the abdominal wound closed, and patient put to bed. Thirty-six hours later the angle of the wound again began to discharge fecal matter. The family declined to have further interference, and, as we felt that there were still open spots of intestine that would eventually slough, we did not insist very strongly upon it. She died ten days after the last operation. Drs. Hood and McCalla assisted at both operations.

SUPRA-PUBIC CYSTOTOMY.

Dr. W. S. Elkin presented a patient upon whom he had performed supra-pubic cystotomy two weeks previous.

The patient was a man thirty-five years of age, weak and very much emaciated. He had consulted him in reference to a cystitis that had been troubling him for the past six or seven years. Upon examination, a stone of considerable size was detected, and supra-pubic cystotomy advised. June 5th, 1890, at Providence Infirmary, the operation was performed and a phosphatic stone larger than a hen's egg, and weighing 700 grs., was removed.

The operation was done under strict antiseptics. After washing out the bladder thoroughly with a boracic acid solution (10 grs. to the ounce), an empty gum bag was introduced into the rectum, and this, distended with 14 ounces of water; six ounces of boracic acid solution was then injected into the bladder and held in by a rubber tube tied tightly around the penis. By this means the bladder was brought well above the brim of the pelvis, pushing up the peritoneum, which was not seen during the operation. A vertical incision, beginning three inches above the symphysis pubis, and extending a little below the upper border of the pubic bone, was made through the skin and superficial fascia down to the linea alba.

The linea alba, transversalis, fascia and cellular tissue beneath were then divided, exposing the bladder, which was seized by a tenaculum and two strong silk ligatures passed through its walls on either side of the instrument. The tenaculum was then removed, and the bladder pulled forward and opened between the ligatures. The stone was quite large, of soft phosphatic character, and in attempting to remove it with a pair of stone forceps, through as small an opening as possible, it crushed.

The large fragments were removed with the forceps, and the debris washed out with warm water, by turning the patient on his side and introducing the nozzle of a fountain syringe through the opening into the bladder. Water was also injected into the bladder through a catheter in the urethra. After the bladder had been thoroughly cleansed of all the fragments, a large rubber catheter was introduced through the opening, which acted as a siphon in conveying the urine into a basin placed on the bed by the side of the patient. The wound was then dressed with iodoform gauze and the dressing completed with absorbent cotton and a flannel bandage.

The catheter acted nicely, producing no irritation and draining the bladder so perfectly for the first forty-eight hours that it was not necessary to change the dressing more than twice during this time.

On the morning of the fourth day the drainage tube was removed and the urine allowed to escape through the wound on the dressings, which were changed as often as they became saturated. After the sixth day the patient was more or less able to control his urine, and void it voluntarily, a part of which began to flow through the urethra.

Two weeks after the operation the patient returned to his home in an adjoining county with the wound almost entirely healed, and only a few drops of urine escaping through the opening during micturition.

Dr. Elkin also presented the specimens of two ovarian tumors that he had removed from a patient a few hours previous. One was larger than a child's head, the other the size of a big orange. They were both firmly imbedded in the pelvic cavity, presenting upon examination a hard, immovable mass extending six or eight inches above the brim of the pelvis. The left and larger tumor had a short, broad pedicle, and was bound down by firm and numerous adhesions, and was with much difficulty removed. The right was more easily taken out. The patient, who was already quite weak and anæmic, bore the operation badly, although it lasted only an hour, and there was very little loss of blood. On account of the many adhesions, a drainage tube was used. Three hours after the operation it was discovered that considerable hemorrhage was taking place. The abdomen was reopened, cleansed with hot water, and the bleeding points, which proved to be an oozing from some of the adhesions, was stopped by means of compression with sponges wrung out of hot water. The hemorrhage was easily controlled, and although not enough loss of blood had taken place to produce a fatal result, it augmented greatly the amount of shock. (Dr. Elkin informs your reporter that this patient died the following day from the shock of the operation.)

